# A rare case of right renal ectopia with ureteral triplication in a 37-year-old male: a case report

**DOI:** 10.11604/pamj.2023.45.169.38444

**Published:** 2023-08-18

**Authors:** Sejal Sanjeev Joshi, Avinash Parashuram Dhok, Kajal Ramendranath Mitra, Prashant Madhukar Onkar

**Affiliations:** 1 Department of Radiodiagnosis, NKP Salve Institute of Medical Sciences and Lata Mangeshkar Hospital, Nagpur, India

**Keywords:** Ureteral triplication, renal ectopia, urography computed tomography, Wolffian duct, case report

## Abstract

Ureteral triplication is one of the least encountered congenital malformations of the upper urinary tract. We report a case of a 37-year-old male patient with right renal ectopia with triplication of the ureter which was diagnosed via computed tomography (CT) urography. This is an intriguing example because, as we discovered after reviewing the literature, the presentation is distinctive.

## Introduction

One of the most remarkable and uncommon anomalies found in the upper urinary tract is a triplication of the ureter. It was first described by Wrany in 1870. It is noted more on the left than the right side and more frequently in women than men. After a complete review of the clinical literature, one comes across the fact that only 100 such cases have been reported to date [1]. We present this case of right renal ectopia with triplication of the ureter which was incidentally detected on CT urography. According to the literature, this kind of renal and ureteral malformation has not been described previously.

## Patient and observation

**Patient information:** a 37-year-old male patient presented to the surgical outpatient department (OPD) with vague abdominal pain often localized to the right lumbar and iliac region. The patient had no history of any co-morbid conditions or surgical history. The patient did not give a history of any previous hospital admissions, any psychiatric disorder, or a history of similar complaints in the family members.

**Clinical findings:** after clinical examination, the patient turned out to have diffuse abdominal pain predominantly in the right lumbar region which was insidious in onset, intermittent, did not radiate elsewhere, and suppressed on symptomatic treatment. The patient did not have any tenderness, guarding, rigidity, or any palpable mass in the right iliac or lumbar region.

**Timeline of the current episode:** he complained that he had been experiencing similar pain since childhood, on and off; and had not attempted to visit any clinic as he hailed from a rural setting. The patient was informed about taking ayurvedic medication for pain relief and he was unable to point out any specific event that triggered the pain.

**Figure 1:**
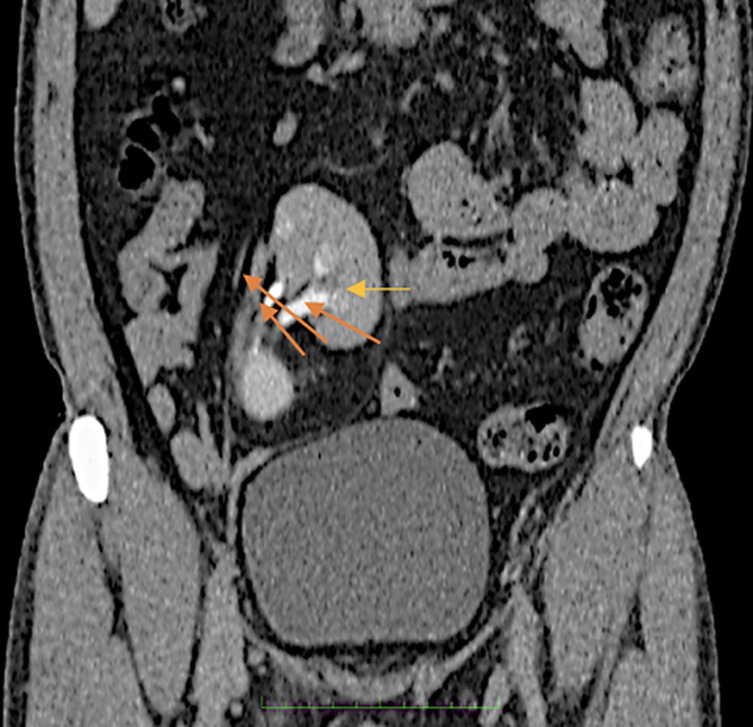
post-contrast coronal plane image of computer tomography urography showing right renal ectopia (yellow arrow) with triplication of the proximal ureter (orange arrows)

**Figure 2:**
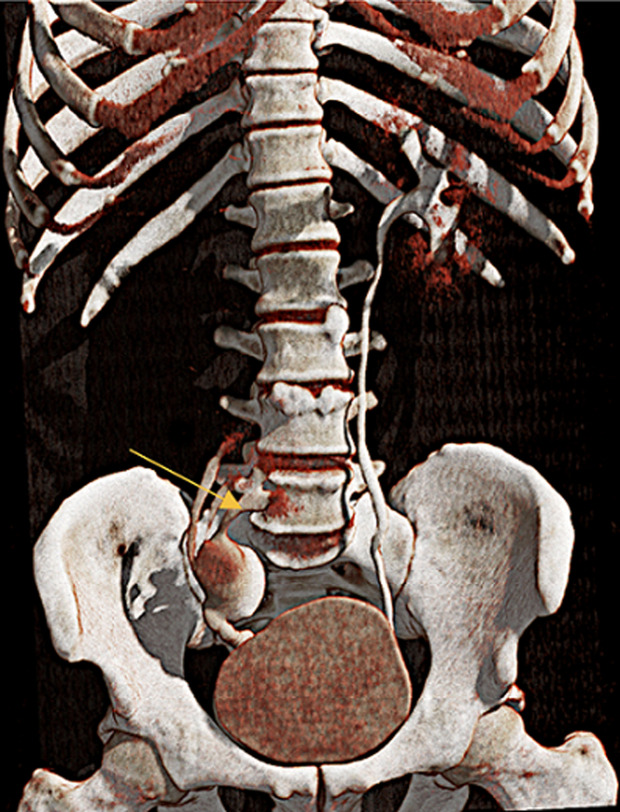
3D volume-rendered reconstruction of the post-contrast coronal image of computer tomography urography showing triplication of ureter combining to form a fusiform distal ureter

**Diagnostic assessment:** upon admission, the patient was advised ultrasonography of the abdomen and pelvis which revealed a small-sized ectopic right kidney measuring approximately 6 x 4 cm with hilum facing laterally. For further evaluation, the patient was advised of CT urography. On the plane, multidetector computed tomography (MDCT) of kidney, ureter, and bladder, right kidney ectopia was noted with no associations with renal or ureteric stones or hydronephrosis. The right kidney was slightly right of midline at the level of the L4-L5 vertebra with the hilum facing right laterally. The contrast was given and the nephrogenic phase revealed lateral facing hilum, 10 min delayed phase of the contrast-enhanced CT, triplication of the proximal ureter was noted which was fused at the level of the upper border of the S1 vertebra and drained into the bladder via the normal right vesicoureteric junction (Figure 1). Three-dimensional volume-rendered reconstruction of the delayed phase scan showed triplication of the ureter combining to form a fusiform mid and distal ureter draining into a single orifice (Figure 2). No abnormalities were associated with the left kidney. Urine analysis, complete blood count (CBC), blood urea nitrogen (BUN), and serum creatinine were within normal limits.

**Diagnosis:** upon contrast-enhanced CT imaging of the kidney, bladder, and ureters, a diagnosis of right renal ectopia with triplication of ureter was made using the plane scan, post-contrast nephrogenic phase and 10 min delayed scan of the CT urography.

**Therapeutic interventions:** the patient was managed conservatively and was prescribed a combination drug of drotaverine and aceclofenac for symptomatic relief as and when needed and was advised to adequate hydration.

**Follow-up and outcome of interventions:** he was asked to follow up in ultrasonographic OPD if and when new symptoms arose like fever, extreme flank pain, hematuria, and pyuria.

**Patient perspective:** patient expressed that he would comply with the follow-up.

**Informed consent:** written informed consent was obtained from the patient for participation in our study.

## Discussion

A seldom-encountered congenital anomaly of the urinary system i.e. ureteral triplication develops from the Wolffian duct around the 5^th^ week of embryonic development [2]. Near the fourth week of gestation, the ureteric bud arises from the Wolffian duct which later forms the ureter. The distal end, between the sixth to eighth weeks of embryonic growth, forms the renal pelvis and calyceal system which occurs cranially and dorsally at the same time [1]. In ureteral triplication, the ureteral buds split early to join the metanephros or emerge independently from the mesonephric duct [2]. This extremely unusual congenital malformation, known as triplicated ureter was first identified by Wrany in 1870 and was later documented for the first time in the literature by Lau and Henline in 1931 [3,4].

Ureteral triplication has four different kinds, according to Smith *et al*. [5]. Type 1: complete ureteral triplication (35%), this type being, three distinct ureters arising from the kidney ending into three different orifices, each of which drains into the bladder or another part of the urogenital tract. Type 2: incomplete triplication (21%). In this type, the kidney produces three ureters, which unite to form two ureteric orifices, two of which unite somewhere along the course. Type 3: trifid ureter (31%), with all three ureters uniting just proximal to a single draining orifice in the bladder. Type 4: double ureter (13%). In this type, two ureters arise from the kidney, one splits and forms an inverse Y bifurcation, draining into three orifices [6]. Although presenting with no specific symptoms, it can occasionally cause discomfort or pain during micturition and incontinence. It may also be the root cause of multiple and recurring urinary tract infection (UTI) episodes [1].

According to Perkins *et al*. ureteral triplications are most frequently accompanied by duplication of the contralateral ureter (37%), ectopic ureteral insertion (28%), and renal dysplasia (8%) [7]. Other pathological conditions associated with ureteral triplication include vesicoureteral reflux, ureteroceles, and pelvis ureterostenoma [2]. The process of failure of adequate cephalic migration of the ureteric bud and metanephric blastema from the pelvis to the flank, during the 5^th^-9^th^ weeks antenatally, results in congenital renal malformations in the position, which is renal ectopia [7,8]. Diagnostic tools for the diagnosis will include ultrasonography, intravenous urography, CT urography, retrograde pyelography, magnetic resonance urography (MRU), and cystoscopy [9]. Our case encompasses the anomaly of both the migration of the metanephric blastema and the splitting of the distal end of the ureteric bud.

## Conclusion

One might fail to diagnose ureteral triplication clinically, due to a lack of specific clinical signs and hence radiological examination remains the mainstay of diagnosis. These anomalies must be documented, as case-specific management on the off chance of genitourinary system pathologies are affected by them. Here we identified a case of ureteral triplication combined with right renal ectopia. Three renal pelvis leading to three different ureters. The three ureters joined together to form a cystically dilated distal ureter before draining into the bladder via a single orifice, which was similar to type III of ureteral triplication as described by Smith *et al*. [5]. To our knowledge, this case has not been described previously.

## References

[1] Li J, Hu T, Wang M, Chen S, Huang L (2004). Ureteral triplication: the first report in China. J Pediatr Surg.

[2] Xu Z, Li Z, Wang D, Deng G, Su C, Pan J (2009). Ureteral triplication combined with right renal ectopia and ureteral cyst. Urol Int.

[3] Babu M, Bansal D, Mehta S, Pillai B, Krishnamoorthy H (2017). Ureteral Triplication: A Rare Congenital Anomaly. Urol Nephrol Open Access J.

[4] Lau FT, Henline RB (1931). Ureteral anomalies: report of a case manifesting three ureters on one side with one ending blindly in an aplastic kidney and a bifid pelvis with a single ureter on the other side. Journal of the American Medical Association.

[5] Smith I (1946). Triplicate ureter. Br J Surg.

[6] Spangler EB (1963). Complete Triplication of the Ureter. Radiology.

[7] Perkins PJ, Kroovand LR, Evans AT (1973). Ureteral triplication. Radiology.

[8] Gutiérrez DM, Rodríguez DF, Guerra JC (2013). Renal anomalies of position, shape and fusion: radiographic analysis. Revista de la federacion ecuatoriana de radiologia.

[9] Gupta S, Vaishnav A, Pal DK (2019). A Case Report of Ureteral Triplication with Contralateral Duplication-A Rare Anomaly with Difficult Diagnosis and Treatment. Journal of Clinical & Diagnostic Research.

